# Critical Location Spatial-Temporal Coverage Optimization in Visual Sensor Network

**DOI:** 10.3390/s19194106

**Published:** 2019-09-23

**Authors:** Yonghua Xiong, Jing Li, Manjie Lu

**Affiliations:** 1 School of Automation, China University of Geosciences, Wuhan 430074, China; lijing0966@163.com (J.L.); 13257163092@163.com (M.L.); 2 Hubei Key Laboratory of Advanced Control and Intelligent Automation for Complex Systems, Wuhan 430074, China

**Keywords:** visual sensor network, spatial-temporal coverage, sensor node scheduling, two-phase coverage-enhancing method

## Abstract

Coverage and network lifetime are two fundamental research issues in visual sensor networks. In some surveillance scenarios, there are some critical locations that demand to be monitored within a designated period. However, with limited sensor nodes resources, it may not be possible to meet both coverage and network lifetime requirements. Therefore, in order to satisfy the network lifetime constraint, sometimes the coverage needs to be traded for network lifetime. In this paper, we study how to schedule sensor nodes to maximize the spatial-temporal coverage of the critical locations under the constraint of network lifetime. First, we analyze the sensor node scheduling problem for the spatial-temporal coverage of the critical locations and establish a mathematical model of the node scheduling. Next, by analyzing the characteristics of the model, we propose a Two-phase Spatial-temporal Coverage-enhancing Method (TSCM). In phase one, a Particle Swarm Optimization (PSO) algorithm is employed to organize the directions of sensor nodes to maximize the number of covered critical locations. In the second phase, we apply a Genetic Algorithm (GA) to get the optimal working time sequence of each sensor node. New coding and decoding strategies are devised to make GA suitable for this scheduling problem. Finally, simulations are conducted and the results show that TSCM has better performance than other approaches.

## 1. Introduction

A visual sensor network (VSN) is commonly comprised of a large number of sensor nodes with limited energy and has been widely applied in many areas such as environment monitoring, traffic surveillance, physical security and so forth [1,2,3].

In many surveillance scenarios, such as battle field, there are some known critical locations where the events of concern are expected to occur. A common goal in such applications is to use sensor nodes to monitor these critical locations with sufficient quality of surveillance with lifetime constraint. However, due to the limited energy of sensor node, it may not be possible to meet both coverage and lifetime requirements. Therefore, how to schedule sensor nodes reasonably to maximize the spatial-temporal coverage of the critical locations and ensure the specified network lifetime is of great research significance.

**Figure 1:**
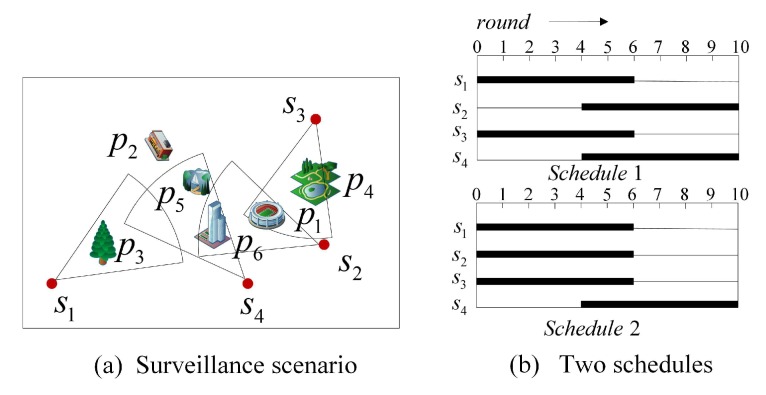
A surveillance scenario in a virtual sensor network (VSN).

Figure 1a illustrates a surveillance scenario in a visual sensor network. We assume that four sensor nodes $s_{1}∼s_{4}$ are randomly deployed to monitor six critical locations $p_{1}∼p_{6}$ for 10 h (Figure 1a). According to the importance of each critical location, the set $w=\{w_{1},w_{2},w_{3},w_{4},w_{5},w_{6}\}$ is used to represent the important factors of the critical locations, in which each important factor is a random value from 0 to 1. It is supposed that the important factor set of the critical locations is w={0.1,0.2,0.1,0.15,0.25,0.2}. Each critical location is modeled as a target, and then the critical location spatial-temporal coverage problem can be regarded as discrete target coverage problem. Different from traditional target coverage, the spatial-temporal coverage of a critical location is defined as the product of the important factor of the location and the length of time during which the location is covered [4]. Since the battery life of each sensor node can only last for 6 h, the network coverage and lifetime requirements can not be satisfied at the same time. Now, the spatial-temporal coverage optimization problem is how to schedule sensor nodes to meet the maximum global spatial-temporal coverage. There are two different schedules in Figure 1b, based on the spatial-temporal coverage metric, the spatial-temporal coverage in Schedule 1 can be calculated as $w_{1}\times 10+w_{2}\times 0+w_{3}\times 6+w_{4}\times 6+w_{5}\times 6+w_{6}\times 6=0.1\times 10+0.2\times 0+0.1\times 6+0.15\times 6+0.25\times 6+0.2\times 6=5.2$, and the spatial-temporal coverage in Schedule 2 is 5.6. Obviously, Schedule 2 is better than Schedule 1 for maximizing the spatial-temporal coverage. The results show that different schedules have a great influence on the spatial-temporal coverage.

In this paper, our goal is how to schedule sensor nodes to maximize the total spatial-temporal coverage with constrained network lifetime, to improve the quality of network monitoring. Due to the limited field-of-view (FOV) of sensor nodes, it is almost inevitable that some critical locations cannot be covered by any sensor node with random deployment. Based on the definition mentioned above , the spatial-temporal coverage of a critical location is determined by the number of sensor nodes covering the critical location and the effective time for the critical location to be covered. Thus, we propose a TSCM to maximize the total spatial-temporal coverage. In TSCM, two optimization models are established, one of which is for maximizing the number of critical locations and the other is for maximizing the spatial-temporal coverage of critical locations with constrained network lifetime. Then, in the first phase, the goal of PSO algorithm is to optimize the sensing directions of sensor nodes, so that sensor nodes can cover more critical locations. And in the second phase, a GA with new coding and decoding strategies is applied to schedule sensor nodes to get the optimal working time sequence of each sensor node, which contributes to enhancing the spatial-temporal coverage. Finally, the experimental results show that TSCM has better performance in aspect of the spatial-temporal coverage.

The rest of paper is organized as follows. Related works are summarized in Section 2. Section 3 introduces the network model and problem formulation. Section 4 describes the proposed method. Simulation results for evaluating the proposed method are presented in Section 5. Finally, Section 6 describes the conclusion of the work.

## 2. Related Works

The coverage and network lifetime issues are basic problems in VSN [5,6]. Some solutions have been proposed to enhance the network coverage or the network lifetime, including deterministic deployment [7,8], sensing range or direction adjustment [9,10,11,12], sensor node scheduling [13,14] and mobile strategy [15,16]. In these strategies, sensor node scheduling is a major technique which can better weigh the network coverage and lifetime.

In the current research works, the maximum target coverage with a minimum number of sensor nodes is an important issue in directional sensor networks. In order to improve the coverage, three local coverage optimization algorithms are proposed to address the coverage problem in Reference [9]. To tackle the issue that how to select the minimum number of sensors to guarantee the full-view coverage for the given region of interest, the authors of Reference [10] propose two different deployment strategies for camera sensor networks. In Reference [11], the authors design distributed clustering and target coverage algorithms to address the problem in an energy-efficient way. In order to extend the network lifetime while satisfying the coverage requirement, the authors of Reference [12] propose an improved genetic algorithm based scheduling for wireless sensor networks. However, in mission-driven applications, the network has constrained lifetime and the objective is to improve the network coverage while meeting the lifetime constraint [17,18,19]. Thus, according to different application scenarios, the goals of the sensor node scheduling can be roughly divided into the following two categories: the network lifetime extension with coverage requirement and the network coverage maximization with a lifetime requirement [20,21].

In terms of sensor node scheduling for the network lifetime extension with coverage requirement, many works focus on how to schedule the directions of sensor nodes into multiple subsets [13,14,22,23] or select a minimum subset of sensor nodes [24,25,26] and each subset that satisfies the coverage requirement performs monitoring task in turns. In Reference [23], the authors address the scheduling problem as a Maximum Set Covers problem and propose a greedy algorithm and a genetic algorithm to obtain the optimal subsets for prolong the network lifetime while ensuring the full target coverage. Based on Reference [23], the authors of [14] also consider the network connectivity and present a greedy algorithm to solve the network lifetime extension problem. Considering the importance of different targets in realistic monitoring scenarios, a learning automata-based algorithm is presented in Reference [24] to select a minimum subset to be activated successively.

However, the problem of how to schedule sensor nodes to maximize the network coverage with lifetime constraint has not been well investigated. This problem was first studied in Reference [27] and a centralized heuristic algorithm and a distributed parallel optimization protocol are proposed to schedule sensor nodes’ activity after they have been deployed. In Reference [4], each critical location is modeled as a discrete target and then a distributed algorithm is proposed to schedule sensor nodes with the objective of maximizing the spatial-temporal coverage while meeting the network lifetime requirement. The sensing models in these above works are all based on traditional omni-directional sensor network, but they are not applicable in visual sensor network. The authors of Reference [28] consider the spatial-temporal coverage with 3D sensing model and propose a distributed sensor node scheduling to obtain the network spatial-temporal coverage optimization.

The sensor node scheduling problem has been proved as NP-hard [29], but the existing methods focus on a finite number of solution spaces and cannot truly achieve global optimization.

Our main contributions can be summarized as follows.

(1) We study how to deal with scheduling sensor nodes for spatial-temporal coverage problem in visual sensor networks, and a TSCM to maximize the spatial-temporal coverage is designed.

(2) We establish two optimization models in TSCM, one of which is for maximizing the number of critical locations and the other is for maximizing the critical location spatial-temporal coverage with constrained network lifetime.

(3) We propose new coding and decoding strategies to make GA suitable for the scheduling problem aiming at maximizing the spatial-temporal coverage under the requirement of the network lifetime.

## 3. Network Model and Problem Formulation

### 3.1. Network Model

To simplify the problem analysis, our discussion is built upon the following assumptions:

(1) All sensor nodes are homogeneous, including the same initial battery, angle of view (AoV), sensing radius and communication ability.

(2) All sensor nodes could guarantee the network connectivity.

We consider a visual sensor network with *n* sensor nodes $S=\{s_{1},\ldots ,s_{i},\ldots ,s_{n}\}$ randomly deployed to monitor *m* critical locations $P=\{p_{1},\ldots ,p_{j},\ldots ,p_{m}\}$ in a 2D area with a size of I×J. We use a common 2D standard model (Figure 1a) for sensor node $s_{i}$ expressed by 5-tuple:

(1) $$s_{i}=\left(\alpha_{i},\varphi_{i},\left(x_{i},y_{i}\right),t_{i},R\right)$$

where $\left(x_{i},y_{i}\right)$ is the coordinate of the sensor node, $\alpha_{i}$ is the angle of view (AoV), and R is the sensing radius. In addition, $\vec{V}$ is the working direction.

**Figure 2:**
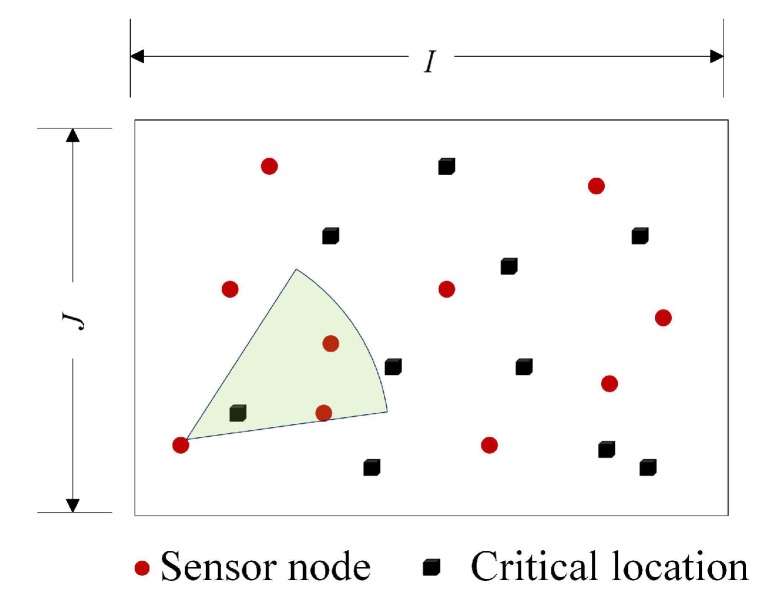
Network Model.

**Figure 3:**
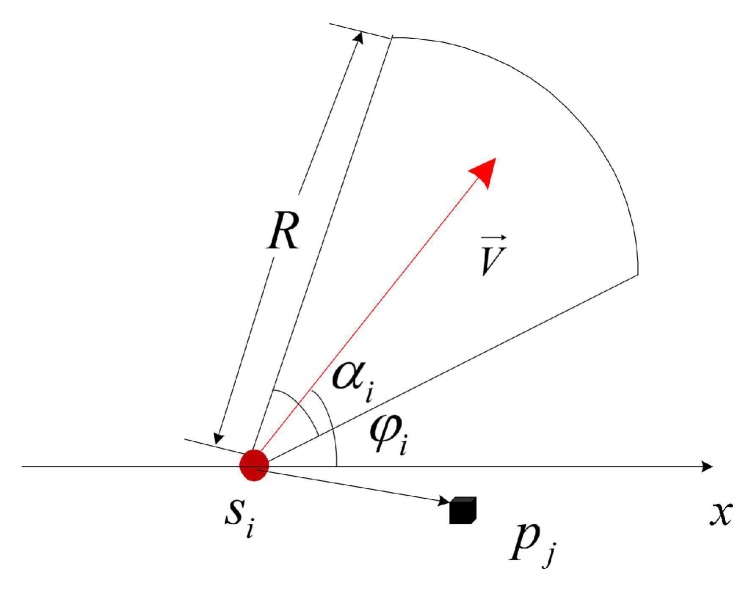
Sensing model.

Figure 2 shows the coverage situation of a critical location in monitoring area, it can be observed that the critical location $p_{j}$ can be covered by the sensor node $s_{i}$ if it satisfies the following two conditions (Figure 3):

(1) The distance $d_{ij}$ between $s_{i}$ and $p_{j}$ must be smaller than or equal to the sensing radius *R*,

(2) $$d_{ij}=\sqrt{{\left(x_{i}-x_{j}\right)}^{2}+{\left(y_{i}-y_{j}\right)}^{2}}\leq R$$

(2) The absolute included angle between and working direction must be smaller than or equal to the half of the AoV,

(3) $$\varphi_{i}=\arccos\frac{\vec{s_{i}p_{j}}\cdot \vec{V}}{\left|\vec{s_{i}p_{j}}\right|}\leq \frac{\alpha_{i}}{2}$$

### 3.2. Problem Formulation

#### 3.2.1. Definitions

**Definition** **1.** *A variable matrix $D=\left[d_{ij}\right]\left(i=1,2\;\ldots \;n,j=1,2\;\ldots \;m\right)$ represents the coverage relationship between the sensor nodes and the critical locations which can be given by*

(4) $$d_{ij}=\left\{\begin{array}{cc} 1 & p_{j}\;coverd\;by\;s_{i}\\ 0 & otherwise \end{array}\right.$$

**Definition** **2.** *The coverage state of critical location $p_{j}$ can be given by*

(5) $$u_{j}=\left\{\begin{array}{cc} 1 & \sum_{i\in S}d_{ij}\neq 0\\ 0 & otherwise \end{array}\right.$$

*If target $p_{j}$ is covered by at least one sensor node, the value of $u_{j}$ is 1, otherwise, the value is 0.*

**Definition** **3.** *Assume that the required network lifetime L is divided into N rounds and the duration time of each round is l. A variable matrix $x_{ik}\left(i=1,2\ldots n,k=1,2\ldots N\right)$ indicates whether or not the sensor node $s_{i}$ is active in k-th round which can be given by*

(6) $$x_{ik}=\left\{\begin{array}{cc} 1 & s_{i}\;is\;active\;in\;k\text{-}th\;round\\ 0 & otherwise \end{array}\right.$$

Then, the coverage state of critical location $p_{j}$ in *k*-th round can be represented by the binary variable $v_{jk}$. If critical location $p_{j}$ is covered by at least one sensor node in *k*-th round, the value of $v_{jk}$ is 1, otherwise, the value of $v_{jk}$ is 0. It can be expressed as follows:

(7) $$v_{jk}=\left\{\begin{array}{cc} 1 & \sum_{i\in S}x_{ik}\times d_{ij}\neq 0\\ 0 & otherwise \end{array}\right.$$

#### 3.2.2. Problem Formulation

The spatial-temporal coverage of a critical location is defined as the product of the important factor of the location and the length of time during which the location is covered, and then within the specified network lifetime, the total spatial-temporal coverage can be calculated as the sum of each critical location’s spatial-temporal coverage. Assume that the subset $X_{k}\subset S$ is activated in *k*-th round, then for any critical location $p_{j}$, the value of spatial-temporal coverage during the *N* rounds duty process can be calculated as follows:

(8) $$Cov\left(p_{j}\right)=\sum_{k\in N}w_{j}\times v_{jk}\times l$$

Thus, the total spatial-temporal coverage is expressed by:

(9) $$\bar{C}=\sum_{j\in P}Cov(p_{j})$$

Now, the scheduling optimization problem is

(10) $$\max\;\bar{C}$$

s.t.

(11) $$\sum_{k=1}^{N}x_{ik}\times l\leq t_{i}$$

(12) $$\sum_{i=1}^{n}x_{ij}\geq 1$$

In this expression, (11) means that, for each sensor node, its total activation time during the *N* rounds should be less than the survivable time . The constraint (12) indicates that at least one sensor node is in an active state during each round to ensure that the specified network lifetime requirement.

## 4. Two-Phase Spatial-Temporal Coverage-Enhancing Method

Based on the spatial-temporal coverage metric, whether a critical location is covered by sensor nodes is the primary factor determining the spatial-temporal coverage value of it. But the limited FOV and random deployment of sensor nodes would make some critical locations not covered by any sensor node. Thus, we propose a TSCM to maximize the spatial-temporal coverage. In phase one, to maximize the number of critical locations covered by sensor nodes, a particle swarm optimization (PSO) algorithm is employed to adjust the sensing directions of sensor nodes to change the initial coverage relationship between sensor nodes and critical locations gotten by random deployment. In phase two, we apply a genetic algorithm (GA) to schedule sensor nodes to obtain the optimal working time sequence of each sensor node. Then, the spatial-temporal coverage maximization with the network lifetime constraint can be achieved by the operations above mentioned. Algorithm 1 describes the proposed method. 

**Algorithm 1** TSCM
1:Input: Sensor node set *S*, critical location set *P*, round *N*, duration time *l*2:Output: The value of spatial-temporal coverage3:Deploy *S* and *P* randomly4:Go into Phase-I: Adjust sensor nodes’ directions using PSO algorithm such that the number of critical location covered is maximum5:Go into Phase-II: Design sensor node schedule using GA for sensor node scheduling such that the spatial-temporal coverage is maximum


### 4.1. Phase I: Maximize the Number of Covered Critical Locations Based on Particle Swarm Optimization Algorithm

Seen from the Equations (8)–(10), the spatial-temporal coverage value for a critical location is determined by whether it is covered and the effective covered time. But when the sensor nodes is not enough in monitoring area, the limited FOV and random deployment of sensor nodes would make some critical locations not covered by any sensor node. To maximize the number of covered critical locations, we use particle swarm optimization algorithm to optimize the sensing directions of sensor nodes. PSO [30] is a population-based search algorithm, where each particle represents a potential solution. It has the advantage with simple and good convergence so that PSO can be used for a wide variety of design and optimization tasks.

(1) Coding and Initialization 

The PSO algorithm uses the following real number vector to denote the solution to the problem:

(13) $$\beta_{i}=\left(\beta_{i1},\beta_{i2},\ldots ,\beta_{in}\right),1\leq i\leq num$$

where $\beta_{i}$ represents position of *i*-th particle and num is the size of the population, *n* is the number of sensor nodes. All particles are randomly generated in the *n*-dimension space.

(2) Fitness 

Mathematically, fitness is considered as an objective function used to evaluate the particles in a population. In the PSO, a fitness function has a non-negative value. For an individual, the larger the value is, the more suitable the solution is. The objective of the problem is to maximize the number of covered critical locations which is equal to maximize the total important factor. So, we define the fitness of a particle as

(14) $$f=\sum_{j=1}^{m}u_{j}\times w_{j}$$

After calculating the fitness of each particle, the algorithm should select the personal best value $P_{best}$ and the global best value $G_{best}$.

(3) Velocity and Position Update 

The particle position of *i*-th particle is represented as $\beta_{i}$, and its velocity is represented as $\varepsilon_{i}=\left(\varepsilon_{i1},\varepsilon_{i2},\ldots ,\varepsilon_{in}\right)$, that is, the change difference of the sensing directions. Then, the particles are manipulated according to the following two equations [29]:

(15) $$\varepsilon_{id}\left(t+1\right)=\omega \cdot \varepsilon_{id}\left(t\right)+k_{1}\cdot b_{1}\cdot \left(P_{best}-\beta_{id}\left(t\right)\right)+k_{2}\cdot b_{2}\cdot \left(G_{best}-\beta_{id}\left(t\right)\right)$$

(16) $$\beta_{id}\left(t+1\right)=\beta_{id}\left(t\right)+\varepsilon_{id}\left(t+1\right)$$

where $k_{1}$ and $k_{2}$$\left(0\leq k_{1},k_{2}\leq 4\right)$ are learning factors, $b_{1}$ and $b_{2}$ $\left(0\leq b_{1},b_{2}\leq 2\right)$ are random values. The constants $k_{1}$ and $k_{2}$ determine the speed that a particle would accelerate towards the personal best value and the global best value. Usually, $k_{1}$ and $k_{2}$ are equal to 2 [29], but other values can also be taken . Generally speaking, $k_{1}=k_{2}$, and the range is between 0 and 4 [30].

The inertia weight coefficient *w* can be fixed or linear decrease. Researches have shown that bigger inertia weight coefficient contributes global search, while smaller inertia weight coefficient contributes local search. Moreover, the experiments show that when *w* is between 0.4 and 0.9, the PSO algorithm has faster convergence speed, yet, when *w* is over 0.9, it is also easy to fall into local minimum [30]. Therefore, the linear decrement inertia weight coefficient is as follows.

(17) $$w\left(t\right)=w_{\max}-\frac{w_{\max}-w_{\min}}{t_{\max}}\cdot t$$

where $t_{\max}$ is the maximum number of iterations, *t* is the current iteration, $w_{\max}$ and $w_{\min}$ are the maximum and minimum values of the inertia weight coefficient *w*, respectively. In this paper, $w_{\max}$ is 0.9 and $w_{\min}$ is 0.4, as in Reference [30].

(4) Termination Condition 

The termination condition of our PSO is simply checking whether the algorithm has been running for a fixed number of generations. When the algorithm terminates, it will output the optimal sensing directions of the sensor nodes. According to the sensing model, the relationship matrix $d_{ij}$ can be calculated, which is going to be the input of the second phase in our method.

### 4.2. Phase II: Sensor Node Scheduling for Maximizing the Spatial-Temporal Coverage

As forward mentioned, the spatial-temporal coverage value for a critical location is determined by whether it is covered and the effective covered time. To maximize the effective covered time for a critical location, we need to get the optimal working time sequence of each sensor node. GA [31] has several advantages, such as fast convergence and simplicity, and is widely used in sensor network scheduling problems. Thus, we employ a GA to solve the sensor scheduling problem. In GA, each solution is encoded in a finite length string, called an individual, based on certain rules. A fitness function is used to evaluate the fitness of each individual. Three operations are performed on the current population to create the next generation: selection, crossover, and mutation.

(1) Coding and Initialization 

We use a GA based on two-dimensional binary coding to denote the solution to the problem:

(18) $$X_{z}=\left[\begin{array}{ccc} x_{11}^{z} & \ldots & x_{1N}^{z}\\ \ldots & x_{ik}^{z} & \ldots \\ x_{n1}^{z} & \ldots & x_{nN}^{z} \end{array}\right],1\leq z\leq popsize$$

where $X_{z}$ represents *i*-th individual and popsize is the size of the population, *n* is the number of sensor nodes, *N* is the number of rounds. All individuals are randomly generated.

(2) Matrix-based Decoding Algorithm 

The process of decoding involves applying the Algorithm 2 to an individual to generate a schedule. The Algorithm 2 calculates the value in the coverage relationship $d_{ij}$ according to Equations (2) and (3), and then judges the rounds for the covered critical locations.

**Algorithm 2** Matrix-based Decoding Algorithm1: Input: *S*, *P*, *N*, *l*, $X_{m}$2: Output: Set of rounds for covered critical location $L_{j}$3: Initialize $A_{ij}=\left[\;\right]$, $L_{j}=\left[\;\right]$4: Calculate the values in $A_{ij}$ according to Equations (2) and (3)5: for *j*=1,2,...,*m* do6:  for *k*=1,2,...,*N* do7:   for *i*=1,2,...,*n* do8:    $d_{ijk}=d_{ij}\times x_{ik}$9:   end for10:   if $d_{ijk}\geq 1$11:   $L_{j}\leftarrow L_{j}\cup k$12:   end if13:  end for14: end for

For example, the individual randomly generated by genetic algorithm is as follows.

(19) $$X=\left[\begin{array}{cccccccccc} 1 & 0 & 1 & 1 & 1 & 0 & 1 & 0 & 1 & 0\\ 1 & 0 & 1 & 0 & 1 & 1 & 0 & 1 & 1 & 0\\ 0 & 1 & 1 & 1 & 0 & 0 & 1 & 0 & 1 & 1\\ 0 & 1 & 0 & 1 & 1 & 0 & 1 & 1 & 0 & 1 \end{array}\right]$$

According to Figure 1a and Equation (4), we can get the coverage relationship between the sensor nodes and the critical locations:

(20) $$D=\left[\begin{array}{cccccc} 0 & 0 & 1 & 0 & 0 & 0\\ 1 & 0 & 0 & 0 & 0 & 1\\ 1 & 0 & 0 & 1 & 0 & 0\\ 0 & 0 & 0 & 0 & 1 & 1 \end{array}\right]$$

After using the matrix-based decoding method, we can get the set of rounds for each covered critical location $L_{1}=\left[1,2,3,4,5,6,7,8,9,10\right],L_{2}=\left[\;\right],L_{3}=\left[1,3,4,5,7,9\right],L_{4}=\left[2,3,4,7,9,10\right],$$L_{5}=\left[2,4,5,7,8,10\right],L_{6}=\left[1,2,3,4,5,6,7,8,9,10\right]$.

(3) Fitness 

The objective of the problem is to maximize spatial-temporal coverage. So, we define the fitness of a particle as

(21) $$f=\bar{C}$$

According to (8), (9) and (21), the total spatial-temporal coverage for the above example can be calculated as $w_{1}\times 10+w_{2}\times 0+w_{3}\times 6+w_{4}\times 6+w_{5}\times 6+w_{6}\times 10=0.1\times 10+0.2\times 0+0.1\times 6+0.15\times 6+0.25\times 6+0.2\times 10=6$.

(4) Operator Operation 

GA searches in the iterative process of “generating” and “detecting”. It takes the individual in the population as the object, and achieves evolution by basic genetic operator operations. Generally, new populations can be generated by selection, cross, and mutation. Considering the characteristics of 0_1 encoding, we use tournament selection and two-points crossover operation to complete the intersection. Meanwhile, a multi-point mutation operator is designed to carry out the mutation operation process.

## 5. Simulation Results

To reduce the errors caused by randomness, the experimental results take the average of 10 runs. The size of monitoring region is 10×10 , the number of sensor nodes and targets is 10 to 50. The important factor of each target is equal to 1. The sensing range varies from 1 m to 5 m and the ratio of battery/network lifetime is ν that varies from 1/5 to 3/5. We conduct experiments by changing the number of sensor nodes, the number of targets, sensing range and the ratio of battery/network lifetime to compare the performance of TSCM, Distributed ECT [4], STCOS algorithm [26], MinRedancy algorithm [25] and RndScheduling algorithm [19].

**Distributed ECT:** The distributed algorithm uses the point coverage model to model the critical location coverage problem to maximize the effective coverage time by scheduling sensors while meeting the network lifetime requirement.

**STCOS algorithm:** The spatial-temporal coverage optimization scheduling algorithm is an optimization method to find the most desired scheduling scheme of each node according to the relative position of its neighbor’s on-period to maximize the spatial-temporal coverage of the whole network.

**MinRedancy algorithm:** It is a distributed heuristic scheduling algorithm that chooses the sensor who has minimum overlapping area with the determined scheduling sensor node to determine its scheduling preferentially.

**RndScheduling algorithm:** This is a random schedule algorithm. Each node generates a random schedule. Then the redundant nodes are turned off, and only the nodes which can contribute to the coverage will remain power on.

**Figure 4:**
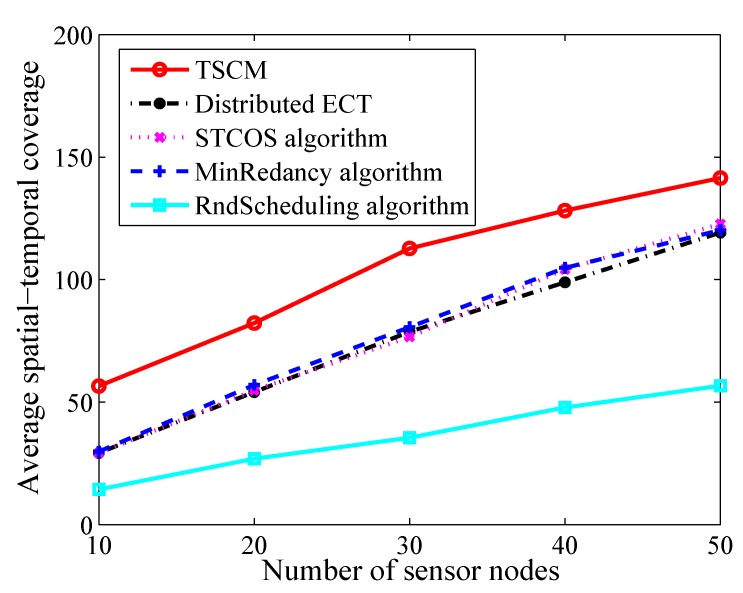
The average spatial-temporal coverage versus different number of sensor nodes.

We now fix the number of targets to 20, sensing range to 5 m and the ratio of battery/network lifetime to 0.2. Figure 4 compares the performance of TSCM ,Distributed ECT, STCOS algorithm, MinRedancy algorihtm and RndScheduling algorithm with changing the number of sensor nodes. The simulation result indicates that increasing the number of sensor nodes contributes to the improvement of the spatial-temporal coverage by all algorithms. Because the more sensors there are, the more targets can be covered, which helps to improve the spatial-temporal coverage. Meanwhile, as the number of sensor nodes increases, the differences between TSCM and other algorithms are gradually narrowed. Compared to Distributed ECT, STCOS algorithm, MinRedancy algorihtm and RndScheduling algorithm, the result shows that the maximum improvement is 48.2%, 48.4%, 47.4%, 74.6% and the average improvement is 30.3%, 29.2%, 28.0%, 66.6% by TSCM, respectively.

**Figure 5:**
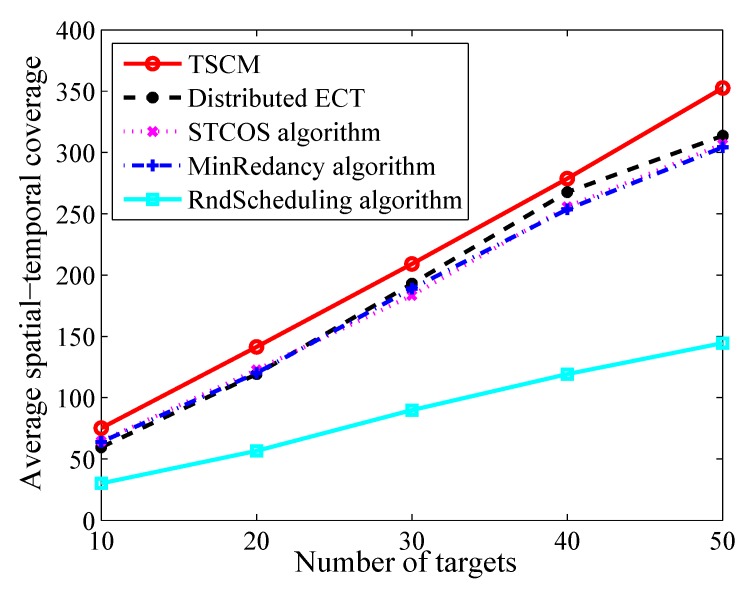
The average spatial-temporal coverage versus different number of targets.

Next, we set the number of sensor nodes at 50, sensing range at 5 m and the ratio of battery/network lifetime at 0.2. Figure 5 compares the performance of TSCM, Distributed ECT, STCOS algorithm, MinRedancy algorihtm and RndScheduling algorithm with changing the number of targets. The result indicates that when the number of targets increases, the number of covered targets will also increase, which contributes to the spatial-temporal coverage. Compared to Distributed ECT, STCOS algorithm, MinRedancy algorihtm and RndScheduling algorithm, the result shows that the maximum improvement is 20.7%, 14.2%, 15.0%, 59.9% and the average improvement is 11.8%, 12%, 12.3%, 58.4% by TSCM, respectively.

**Figure 6:**
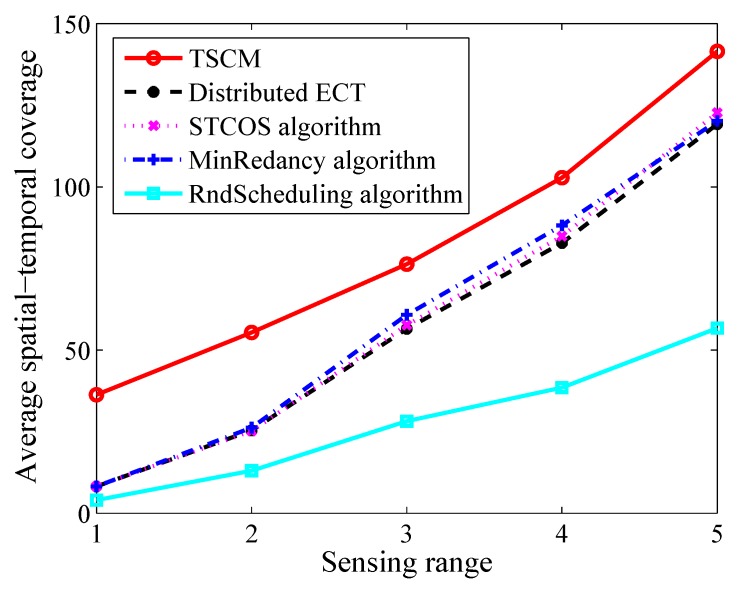
The average spatial-temporal coverage versus different sensing range.

Then, we fix the number of sensor nodes and targets to 50 and 20 respectively, and the ratio of battery/network lifetime to 0.2. The simulation result displayed in Figure 6 shows that when the sensing range in the range of [1, 5], the TSCM can obtain 77.4%, 77.2%, 77.0%, 89% maximum increase and 39.3%, 37.4%, 35.9%, 70.2% average increase, respectively. Moreover, with the increase of sensing range, more targets can be covered, so increasing the sensing range of each sensor node is effective for maximizing the spatial-temporal coverage.

**Figure 7:**
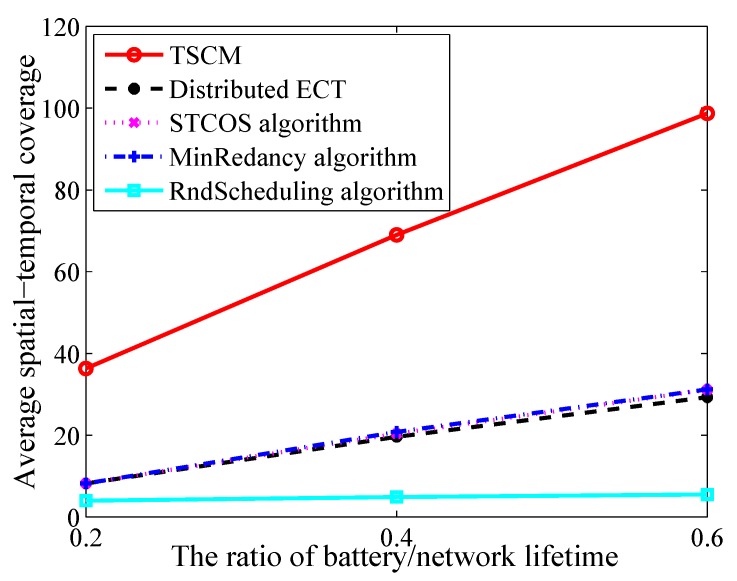
The average spatial-temporal coverage versus different number of targets.

Finally, we set the number of sensor nodes and targets at 50 and 20 respectively, and sensing range at 1 m. The simulation result shown in Figure 7 indicates that increasing the ratio of battery/network lifetime is positive correlation by all algorithms. Because the effective time of covered targets is become longer. Compared to Distributed ECT, STCOS algorithm, MinRedancy algorihtm and RndScheduling algorithm, the result shows that the maximum improvement is 77.1%, 76.8%, 76.8%, 94.1% and the average improvement is 73.3%, 72.2%, 71.8%, 92.0% by TSCM, respectively.

In a word, TSCM has more advantages in spatial-temporal coverage than other four methods in terms of changing the number of sensor nodes, the number of targets, sensing range and the ratio of battery/network lifetime. The results of the comparison are listed in Table 1.

## 6. Conclusions

The coverage and network lifetime are two key issues in visual sensor networks. In this study, we analyze the relationship between the coverage and network lifetime and devise a TSCM to maximize the spatial-temporal coverage within a constraint network lifetime.

To verify the effectiveness of the method, we design and implement simulation experiments by changing the number of sensor nodes, the number of targets, sensing range and the ratio of battery/network lifetime. The results indicate that TSCM is confirmed to exceed Distributed ECT, STCOS algorithm, MinRedancy algorihtm and RndScheduling algorithm for maximizing the spatial-temporal coverage in a situation where the number of sensor nodes is small.

However, more efforts are still needed to further this study and they include considering the network connectivity and coverage redundancy.

## Figures and Tables

**Table 1:** Algorithm Comparison Results.

		Distributed ECT	STCOS	MinRedancy	RndScheduling
Number of sensor nodes	Maximum	48.2%	48.4%	47.4%	74.6%
	Average	30.3%	29.2%	28.0%	66.6%
Number of targets	Maximum	20.7%	14.2%	15.0%	59.9%
	Average	11.8%	12%	12.3%	58.4%
Sensing range	Maximum	77.4%	77.2%	77.0%	89%
	Average	39.3%	37.4%	35.9%	70.2%
The ratio of battery/network liftime	Maximum	77.1%	76.8%	76.8%	94.1%
	Average	73.3%	72.2%	71.8%	92.0%
